# Gender-Associated Impact of Early Leucine Supplementation on Adult Predisposition to Obesity in Rats

**DOI:** 10.3390/nu10010076

**Published:** 2018-01-12

**Authors:** Nora López, Juana Sánchez, Andreu Palou, Francisca Serra

**Affiliations:** Laboratorio de Biología Molecular, Nutrición y Biotecnología (LBNB), University of the Balearic Islands and CIBER de Fisiopatología de la Obesidad y Nutrición (CIBEROBN), E-07122 Palma de Mallorca, Spain; norals@msn.com (N.L.); joana.sanchez@uib.es (J.S.); francisca.serra@uib.es (F.S.)

**Keywords:** leucine supplementation, metabolic imprinting, perinatal nutrition, obesity, biomarkers

## Abstract

Early nutrition plays an important role in development and may constitute a relevant contributor to the onset of obesity in adulthood. The aim of this study was to evaluate the long-term impact of maternal leucine (Leu) supplementation during lactation on progeny in rats. A chow diet, supplemented with 2% Leu, was supplied during lactation (21 days) and, from weaning onwards, was replaced by a standard chow diet. Then, at adulthood (6 months of age), this was replaced with hypercaloric diets (either with high-fat (HF) or high-carbohydrate (HC) content), for two months, to induce obesity. Female offspring from Leu-supplemented dams showed higher increases in body weight and in body fat (62%) than their respective controls; whereas males were somehow protected (15% less fat than the corresponding controls). This profile in Leu-females was associated with altered neuronal architecture at the paraventricular nucleus (PVN), involving neuropeptide Y (NPY) fibers and impaired expression of neuropeptides and factors of the mTOR signaling pathway in the hypothalamus. Interestingly, leptin and adiponectin expression in adipose tissue at weaning and at the time before the onset of obesity could be defined as early biomarkers of metabolic disturbance, predisposing towards adult obesity under the appropriate environment.

## 1. Introduction

Obesity in Western industrialized societies is increasing at an alarming rate [1] and this is not always related to a present unhealthy diet or lifestyle. In this context, early-life nutrition may play an important role as it has been described that specific conditions, foods or environmental inputs at early ages may act as modifiers of metabolic performance and may, for instance, contribute to the implementation of the obese phenotype in adulthood [2,3,4,5,6] in a more or less irreversible manner.

Leucine (Leu) is one of the most abundant essential amino acids in foods, including breast milk, where it can be found forming part of proteins and also as a free, soluble amino acid [7,8], thus, allowing rapid bioavailability in newborn and infant intestinal tracts [9]. Furthermore, Leu contributes to multiple metabolic roles far beyond being a substrate for protein synthesis. Leu has been suggested to influence insulin and glucose homeostasis, as well as adipogenesis and fat accretion [10,11,12]. Substantial experimental evidence supports that Mechanistic Target of Rapamycin Kinase (mTOR) signaling and its downstream effectors may mediate the role of Leu [2,13,14]. One of the first demonstrations on the beneficial role of Leu on body weight and adiposity was carried out in high-fat (HF) fed mice, where adding Leu in drinking water decreased diet-induced obesity and improved plasma glucose and cholesterol levels [15]. Since then, other studies have characterised the effects of Leu supplementation on a number of species, with different doses and protocols for the regulation of food intake, energy balance and glucose homeostasis (see [9,16] for comprehensive reviews). Although beneficial effects have not been clearly established, evidence has shown that leucine supplementation may modulate obesity and improve glucose homeostasis. At present, data seem to confirm that Leu decreases body weight in obese animals, improves glucose metabolism and causes minor effects on lipid metabolism, when the dose in drinking water is around 1.5%, and this is associated with a total Leu intake of two times the standard daily intake [2,17,18]. Furthermore, the effects are more noticeable under a HF diet, whereas in control animals there is no effect at all. In contrast with the above research, a recent series of experiments has demonstrated that short-term removal of dietary Leu may counteract obesity too. The response to removal of Leu is associated with increased β-oxidation, activation of uncoupling protein 1 (UCP1) and diminished lipogenesis in obese animals, which seem mediated by hypothalamic leptin signaling [19,20,21,22,23]. 

Therefore, animal studies are inconclusive in addressing whether diets should be supplemented with Leu or, on the contrary, levels of Leu should be decreased in order to prevent obesity and/or to improve glucose/insulin homeostasis. In this context, we have previously reported that maternal Leu supplementation during lactation may improve body composition in lactating dams, probably mediated by modulation of neuropeptide expression at the hypothalamus level, without compromising the growth of the offspring [24]. However, the long-term effects of maternal Leu supplementation on the long-term health of the offspring have not been previously studied. Thus, the goal of the present study was to analyse the follow up of offspring from Leu-supplemented dams and to assess whether or not the progeny was metabolically better prepared for preventing obesity in adulthood under the nutritional challenge of high-energy dense diets.

## 2. Materials and Methods 

### 2.1. Animals and Experimental Design

Twelve adult virgin female Wistar rats (Charles River Laboratories, Barcelona, Spain) were housed under controlled conditions (22 °C, 12 h light–12 h dark cycle, light on from 08:00–20:00 h) and adapted to a powder standard diet (3.0 kcal/g containing 1.11 g L-leucine/100 g) for four weeks (Diet A04, Panlab, Barcelona, Spain). Then, they were mated and placed in individual cages with free access to water and diet. At day 1 after delivery, the number of pups in each litter was adjusted to 10 per dam (five females and five males when possible) and after that, half of the dams received the control diet (group C) and the other half the same diet supplemented with 2% L-leucine (>99% NT, Sigma–Aldrich, Madrid, Spain) (group L) during lactation.

Weaning was performed on day 21; males and females from offspring were separated, housed in groups of four and fed with the standard diet (without leucine supplementation). To simplify, offspring is named according the dietary treatment followed by the respective mothers (groups C and L), although these animals only received the leucine supplementation during the lactation period. In order to get representative data, the litter of origin was taken into account to select individual animals from offspring and allocate them in the different groups and experimental settings.

To test for susceptibility to obesity in adult life, at 6 months of age, half of the animals (males and females, C and L) were changed to a HF diet (4.7 kcal/g and 45% calories from fat) (D12451, Research Diets, New Brunswick, NJ, USA) and the other half to a HC diet (3.8 kcal/g and 70% calories from carbohydrate) (D12450B, Research Diets, New Brunswick, NJ, USA) for three months, up to day 275 of age on average (referred to as 9 months of age in the following text). Then, animals were sacrificed and samples were obtained from the different groups of both males and females (*n* = 6 in each group, groups C-HC, L-HC, C-HF, L-HF). During adult dietary treatment, a full control offspring group, not challenged with any calorie dense diet, but just regular chow throughout life, would have been a real basal control group. However, this was considered not strictly necessary in our experimental approach, and the application of the principle of replacement, reduction and refinement (3Rs) (Articles 4 and 13 and Annex VI of Directive 2010/63/EU) concerning the use of animals for scientific purposes was prioritised. Furthermore, our main interest was to analyse the response to the excess energy in leucine-treated animals, in comparison with the control offspring group. In this context, offering the same type of diet (either HC or HF) to both control and leucine animals was considered the most suitable experimental setting up. 

### 2.2. Ethics Statement

The animal protocol followed in this study was reviewed and approved by the Bioethical Committee of the University of the Balearic Islands (resolution number 1798 of 18 February 2009). University guidelines for the use and care of laboratory animals were followed.

### 2.3. Follow up and Sample Collection

Body weight and length, food intake and body composition by EchoMRI-700™ (Echo Medical Systems, LLC., Houston, TX, USA) were periodically recorded. Tail blood samples were collected at specific times and serum was obtained after centrifugation at 1000× *g* for 10 min and stored at −20 °C for further analysis. Tissue samples were collected at sacrifice on day 21 (one female and one male from each litter) and at the end of the experiment at 9 months of age. The tissues (mesenteric white adipose tissue and hypothalamus) were removed, immediately frozen in liquid nitrogen and stored at −80 °C until further analysis. The hypothalamus was harvested by using the following landmarks: frontal edge of the optical chiasm, lateral sulci, caudal edge of the mammary bodies, and a depth of 2 mm. All animals were sacrificed under feeding conditions (during the first 2 h of the beginning of the light cycle).

### 2.4. Challenge Tests

An oral glucose tolerance test (OGTT) was performed at the age of 5 months. A load of 1 to 1.5 mL glucose (1.5 g/kg body weight) was orally given to the animals in fasting conditions (during the first 4 h of the beginning of the light cycle in animals deprived of food for 14 h). Blood samples were taken from the tail of animals before the glucose load (time zero) and then at 30, 60, 120, and 180 min thereafter. Plasma glucose was measured with an Accu-check Glucometer (Roche Diagnostics, Barcelona, Spain) and insulin levels were determined using an ELISA kit (DRG Instruments, Marburg, Germany). The homeostatic model assessment for insulin resistance (HOMA-IR) was used to assess insulin homeostasis.

Food preferences were assessed by the two-bottle preference test, at the age of 165 days, as previously described [25]. In brief, rats were simultaneously offered two bottles containing either a Carbohydrate-Rich (CR) liquid diet or a Fat-Rich (FR) liquid diet. The two diets had identical caloric density (2.31 kcal/g) and the following ingredients: for the CR diet, 10 g/100 mL skimmed milk, 40 g/100 mL sucrose, 4 g/100 mL olive oil, and 0.35 g/100 mL xanthan gum (Sigma, Madrid, Spain); and for the FR diet, 10 g/100 mL skimmed milk, 10 g/100 mL sucrose, 17.3 g/100 mL olive oil, and 0.35 g/100 mL xanthan gum. Before performing the test, the rats were habituated over a period of 10 days, with each bottle being given individually on alternate days for 1 h without withdrawing the standard chow diet. The test started 2 days after the adaptation period. Solid food was withdrawn at the beginning of the light phase and two bottles containing preweighed quantities of either the CR or FR diet were placed side-by-side, 4 h after the beginning of the light cycle, for 1 h. The bottles were then reweighed, intake determined and corrected for spillage. Spillage was estimated by weighing small collection plates placed underneath the spout of the bottles. To discard a potential effect of the side, half of the rats had the CR bottle on the left and the FR bottle on the right and this was reversed in the other half. The test was performed on two different days.

### 2.5. Gene Expression

Total RNA was extracted from the hypothalamus and mesenteric white adipose tissue (mWAT) depot by Tripure Reagent (Roche Diagnostic Gmbh, Manheim, Germany) according to the manufacturer’s instructions. Isolated RNA was quantified using a NanoDrop ND-1000 spectrophotometer (NanoDrop Technologies Inc., Wilmington, DE, USA) and its integrity and concentration confirmed using agarose gel electrophoresis.

mRNA expression was determined by Real-time Polymerase Chain Reaction (PCR). In short, total RNA (0.25 μg) (in a final volume of 5 µL) was denatured at 65 °C for 10 min and then reverse transcribed to cDNA using MuLV reverse transcriptase (Applied Biosystems, Madrid, Spain) at 20 °C for 15 min, 42 °C for 30 min, with a final step of 5 min at 95 °C in a 2720 Thermal Cycler (Applied Biosystems, Madrid, Spain). Each PCR was performed from diluted cDNA template, forward and reverse primers (1 μM each), and Power SYBER Green PCR Master Mix (Applied Biosystems, Madrid, Spain). The list of genes analysed and the primers used (Sigma, Madrid, Spain) are described in Table S1. Real-time PCR was performed using the StepOnePlus™ Real-Time PCR Systems (Applied Biosystems, Madrid, Spain) with the following profile: 10 min at 95 °C, followed by a total of 40–50 two-temperature cycles (15 s at 95 °C and 1 min at 60 °C). A melting curve was produced after each run, according to the manufacturer’s instructions, to check for the purity of the products. Values for the threshold (Ct) were determined using instrument software (StepOne Plus Software v2.0) (Applied Biosystems, Madrid, Spain) and the relative gene expression was calculated as a percentage of the group of male control animals; 18S or β-actin were used as a reference.

### 2.6. Morphometric and Immunohistochemical Analysis

Tissue specimens of brain were fixed by immersion in 4% paraformaldehyde in 0.1 M sodium phosphate buffer (PB), pH 7.4. After washing in PB overnight, the samples were dehydrated in a graded series of ethanol and embedded in paraffin blocks for light microscopy using Paraplast Plus^®^ (Sigma, St. Louis, MO, USA). Coronal sections (5 µm thick) from the brain were cut using a microtome and mounted on Super-Frost/Plus slides (Menzel-Gläzer, Braunschweig, Germany). These coronal sections were dewaxed in xylene and rehydrated through with decreased graded series of ethanol and finally were washed with deionized water for 5–10 min, approximately. Consecutive sections were cut, in order to perform morphometric and immunohistochemical analyses separately.

In order to determine area and number of cells of the paraventricular nucleus (PVN), the sections were counterstained with haematoxylin/eosin (Panreac Química S.A, Barcelona, Spain), mounted in Eukitt (Kindler, Germany) on SuperFrost/Plus slides. Subsequently, sections were washed with deionized water, dehydrated with increasing concentrations of ethanol (Panreac Química S.A, Barcelona, Spain) and xylene (Panreac Química S.A, Barcelona, Spain), mounted with Eukitt (Panreac Química SA, Barcelona, Spain) and cover-slipped. Morphometric PVN images from light microscopy (4–5 animals each group) were digitalized using a Zeiss Axioskop 2 microscope, equipped with an AxioCam Icc3 digital camera (Carl Zeiss, S.A, Barcelona, Spain). Boundaries of the PVN were drawn interactively in each haematoxylin/eosin-stained image using AxioVision40V 4.6.3.0 software (Carl Zeiss, Imaging Solutions GmbH, Germany). Neuronal density was calculated by dividing the total number of cells in PVN per surface of the nucleus.

Immunohistochemical revelation of neuropeptide Y (NPY) fibers in PVN was performed with the avidin-biotin peroxidase (ABC) method [26]. Sections were incubated sequentially at room temperature in the following solutions: 0.3% hydrogen peroxide (Panreac Química S.A, Barcelona, Spain) in methanol (Panreac Química S.A, Barcelona, Spain) for 10 min to block endogenous peroxidase; EDTA-based solution (pH = 8–8.2) in microwave oven for 30 min and 20 min on ice for antigen retrieval; 2% goat normal serum (Vector Laboratories, Inc., Burlingame, CA, USA) in 0.1% Triton X-100 (Tris) for 20 min to reduce non-specific background staining prior to incubation with primary antibody (polyclonal anti-NPY antibody produced in rabbit, N9528, Sigma-Aldrich, (St. Louis, MO, USA) 1:200 in Tris for 1 h and 15 min at 37 °C); biotinylated goat antirabbit IgG (Vector Laboratories, Burlingame, CA, USA) 1:200 in Tris for 1 h at room temperature; peroxidase-labelled ABC reagent (Vectastain ABC Kite, Vector) in Tris for 30 min at room temperature and Fast 3,3′-diaminobezidine tablet, DAB (Sigma, St. Louis, MO, USA) in Tris for 3 min in dark room for enzymatic development of peroxidase. Subsequently, slides were washed with deionized water, dehydrated with increasing concentrations of ethanol (Panreac Química S.A, Barcelona, Spain) and xylene (Panreac Química S.A, Barcelona, Spain), mounted with Eukitt (Panreac Química SA, Barcelona, Spain) and cover-slipped.

### 2.7. Statistical Analysis

Results were processed with the SPSS 19 Statistical package for Windows (SPSS, Chicago, IL, USA). The threshold of significance was defined in all the situations as * *p* < 0.05 or ** *p* < 0.01. All data are expressed as the mean ± standard error of mean (SEM).

At weaning and up to HC/HF diet, the statistical analysis was performed by two-way ANOVA, considering sex (S) and leucine treatment (L) as variables to assess differences between samples. Then, the student’s *t*-test was used to compare the treatment differences for each gender.

The statistical analysis of the food preference test consisted of a two-way ANOVA, considering type of bottle (FR vs. CR) and treatment (C vs. L).

At 9 months of age, a three-way ANOVA was used to assess statistical differences between groups. In addition to the above variables (S and L), the dietary intervention (D) in adult life (HC or HF diet) was also considered. When an interaction was found, a two-way ANOVA was then used to assess treatment and/or dietary differences inside each gender, followed by a post-hoc student’s *t*-test.

The analysis of the evolution of body weight and food intake was performed using repeated measures ANOVA and considering sex (S), treatment (C vs. L) and diet (HC vs. HF) along time. Linear relationships between key variables were tested using Pearson’s correlation coefficients.

## 3. Results

### 3.1. Maternal Leu Supplementation during Lactation Promoted Decreased Food Intake in Adult Offspring, Particularly for Carbohydrate Rich Diets, without Changes in Body Weight

**Figure 1:**
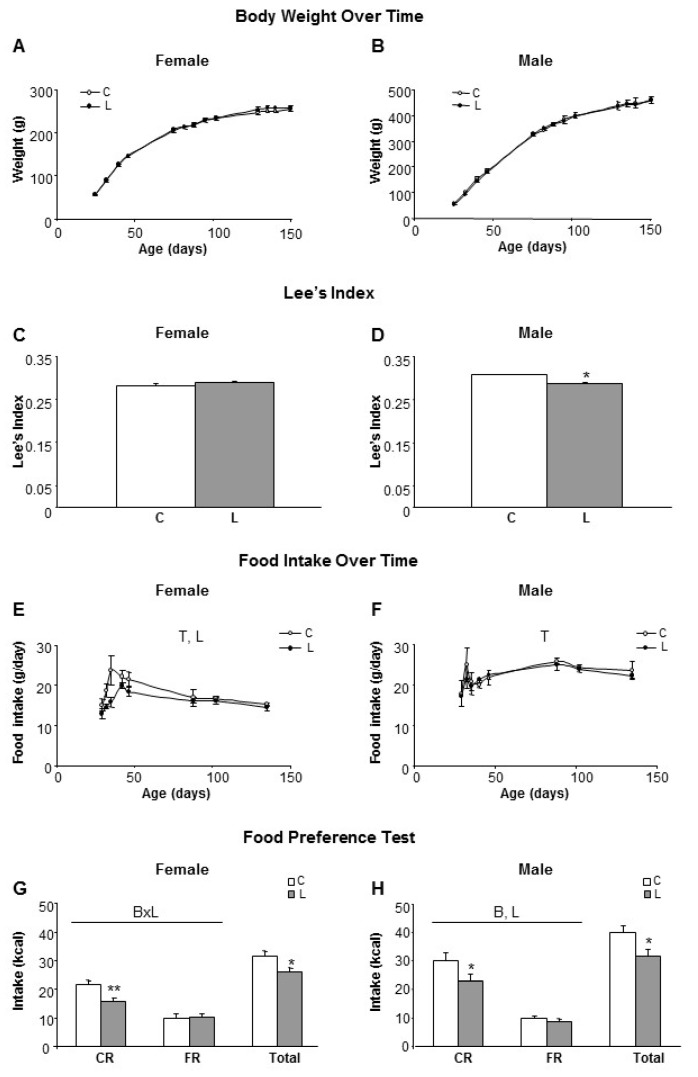
Body weight, food intake and food preferences in offspring of rats supplemented with leucine during lactation. Left side panel (**A**,**C**,**E**,**G**) refers to female and right side panel (**B**,**D**,**F**,**H**) to male siblings. (**A**,**B**) Body weight over time in offspring from dams who received a dietary supplementation of Leu (2%) during lactation, in comparison with non-supplemented controls; (**C**,**D**) Lee’s index measured in adulthood (five months old); (**E**,**F**) Food intake from weaning to adulthood (fed with standard diet); (**G**,**H**) A food preference test was performed (165 days of age) allowing animals free-selection between a fat (FR) or sugary/carbohydrate (CR) enriched liquid diet. Anova: T (time), L (Leu), B (bottle composition). All data represent mean ± SEM. * *p* < 0.05, ** *p* < 0.01.

To assess the potential impact of maternal Leu supplementation on adult health outcomes, the offspring were monitored throughout life. Weaning was performed on day 21 and following this, males and females from offspring were fed ad libitum with the control diet, without Leu supplementation. As stated in the methods section, offspring is named according the dietary treatment followed by their respective mothers (groups C and L). No differences in body weight were found to be associated with maternal treatment up to five months of age (Figure 1A,B). Then, Lee’s index at six months of age indicated that L-males were thinner/smaller than the controls (Figure 1D), whereas no differences were observed within females (Figure 1C). However, L-females showed lower food intakes after weaning, in comparison with their controls (Figure 1E), whereas there were no differences within males (Figure 1F). 

To get further insight, at the level of control of food intake and food preferences, the two-bottle test was used [25,27,28]. Rats were simultaneously offered two bottles containing an identical caloric density (2.31 kcal/g) and either a carbohydrate-rich (CR) liquid diet or a fat-rich (FR) liquid diet, after a training period of 10 days. Both L-females and L-males showed lower total food intakes (17% and 21%, respectively) in comparison with their respective sex-controls, which was mainly reflected in a diminished selection of the carbohydrate-rich diet in both genders (Figure 1G,H). Furthermore, when adjusted to the total energy intake, L-females showed a pattern of lower CR consumption (61% in L vs. 69% in C), that, accordingly, resulted in higher FR consumption (39% in L vs. 31% in C), whereas in males this differential pattern was more attenuated between experimental groups (24% FR and 76% CR in C-males and 27% FR and 73% CR in L-males). Therefore, despite the fact that no impact on the body weight of adult offspring was apparent, the conclusion was that early Leu supplementation in the lactating period altered food intake and dietary preferences in adulthood.

### 3.2. Glucose and Insulin Homeostasis Were Disturbed in Adult Offspring from Leu-Supplemented Dams in a Sex-Specific Manner

The effect of early Leu supplementation in the long-term on glucose/insulin homeostasis was assessed. Glucose and insulin levels at five months of age were not affected by maternal Leu supplementation under feeding conditions. 

**Figure 2:**
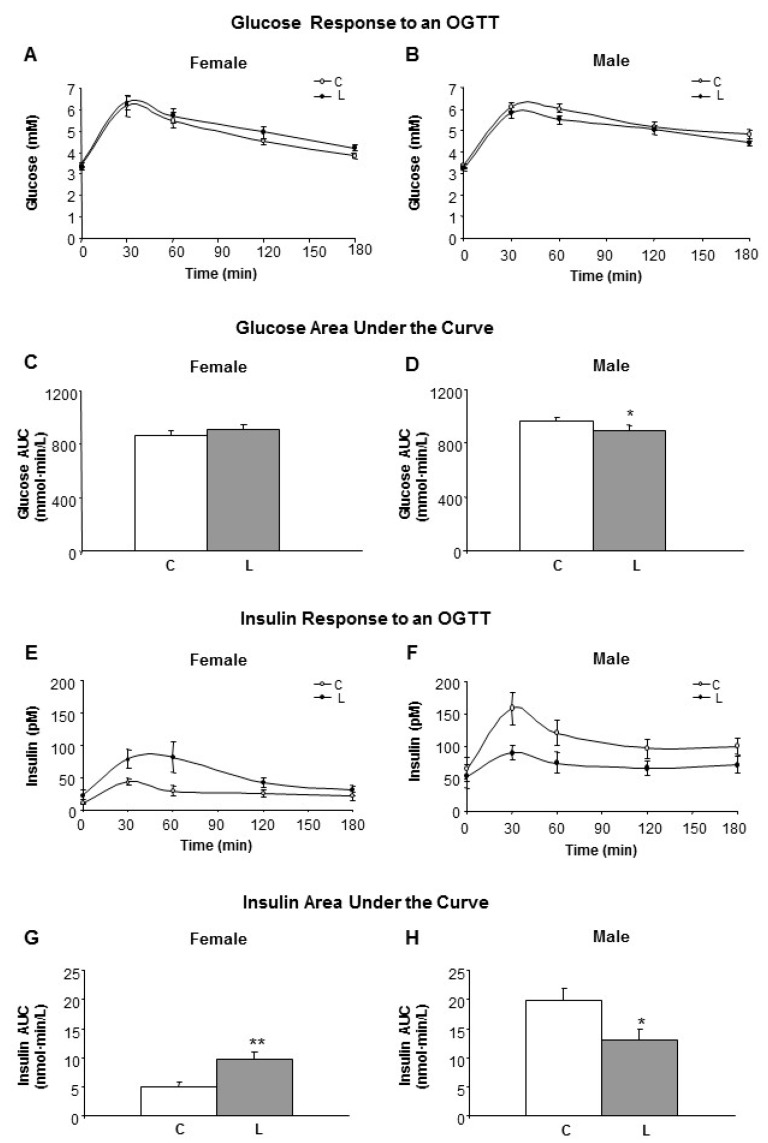
Glucose and insulin response to an oral glucose tolerance test (OGTT). Left side panel (**A**,**C**,**E**,**G**) refers to female and right side panel (**B**,**D**,**F**,**H**) to male siblings. (**A**,**B**) Glucose response to an OGTT in adult offspring from dams who received a dietary supplementation of Leu during lactation, in comparison with non-supplemented controls; (**C**,**D**) Glucose area under the curve (AUC); (**E**,**F**) Insulin levels during OGTT; (**G**,**H**) AUC for insulin. All data represent mean ± SEM. * *p* < 0.05, ** *p* < 0.01.

Under fasting conditions (Time 0 in the OGGT), no differences in either glucose or insulin levels between C- and L-animals were found. However, blood levels of glucose (Figure 2A,B) and insulin (Figure 2E,F) in response to an oral-glucose tolerance test (OGTT) were differentially affected by both early maternal Leu supplementation and offspring gender. 

Therefore, the glucose area under the curve (AUC) was significantly lower in L-males (Figure 2D), whereas no differences were found within females (Figure 2C). Insulin AUC was higher in L-females (Figure 2G) and lower in L-males (Figure 2H), in comparison with the respective controls. Altogether, the HOMA-IR index was kept under control values in L animals (data not shown). In L-males, this was accomplished by a faster removal of serum glucose, associated with a reduced insulin response following the oral glucose load. In contrast, L-females displayed a slower plasma glucose removal using higher insulin levels. Therefore, early Leu supplementation led to higher insulin sensitivity in offspring males but lower insulin sensitivity in the females.

### 3.3. Higher Susceptibility to Adult Obesity Was Promoted by Early Maternal Leu-Supplementation in Female but Not in Male Offspring

**Figure 3:**
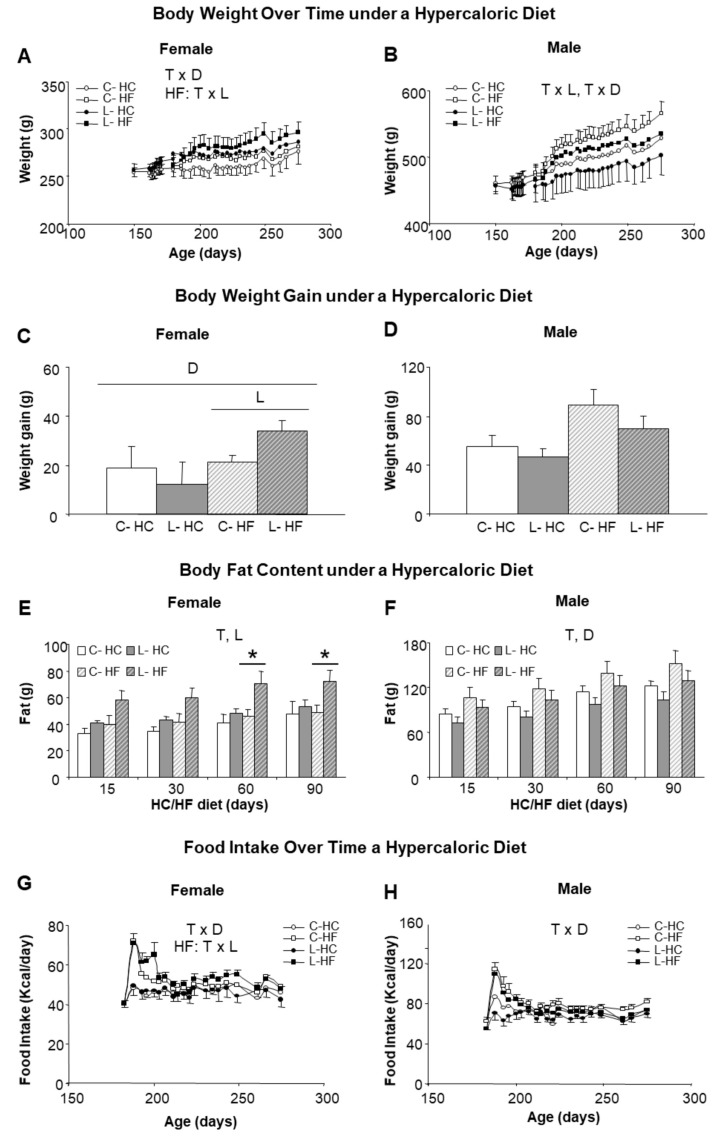
Body weight, body fat content and food intake in response to HF and HC diets. Left side panel (**A**,**C**,**E**,**G**) refers to female and right side panel (**B**,**D**,**F**,**H**) to male siblings. (**A**,**B**) Body weight over time associated with the period (3 months) of feeding with a hypercaloric diet, either high fat (HF) (squares) or high carbohydrate (HC) (circles); (**C**,**D**) Body weight gain after 3 months of feeding a hypercaloric diet; (**E**,**F**) Body fat content accumulated during the period of HF/HC diet; (**G**,**H**) Food intake along the period of HF/HC feeding. Anova: T (time), L (Leu vs. C), D (diet, HF vs. HC). All data represent mean ± SEM, *n* = 6, * *p* < 0.05 C vs. L.

We next investigated the susceptibility to obesity by changing (from six to nine months of age) half of the animals (both males and females and from C and L groups) to a HF (45% calories from fat) and the other half to a HC (70% calories from carbohydrates), keeping protein content at 20% in both diets. Higher body weights were reached with HF diet in comparison with HC diet, suggesting a greater impact of dietary fat than of sucrose. Introduction of the HF diet was associated with noticeable sex- and Leu-specific responses. L-females, either under the HF or the HC diet, showed higher body weights than their corresponding controls, whereas the opposite was found in males (Figure 3A,B). Furthermore, final weight gain was exacerbated under the HF diet in L-females (62% higher than in the controls, C-HF) (Figure 3C); whereas L-males showed a tendency to gain less weight (21% vs. C-HF) (Figure 3D). The analysis of body composition confirmed a higher increase in body fat in L-HF females (47% higher), in comparison with their controls (C-HF) or under the HC diet (Figure 3E) as well as the relative protection in L-males (15% lower) (Figure 3F) under both diets. Differences in fat content in L-HF females appeared at 15 days of the new dietary regime, the period where intake was also higher (Figure 3G), and were statistically significant from day 60 onwards (Figure 3E). In contrast, food intake in L-males was not different from their respective dietary controls throughout the treatment (Figure 3H).

### 3.4. Early Leu Supplementation Was Associated with Long-Term Perturbations of Energy Balance Regulatory Signals in Hypothalamus

**Figure 4:**
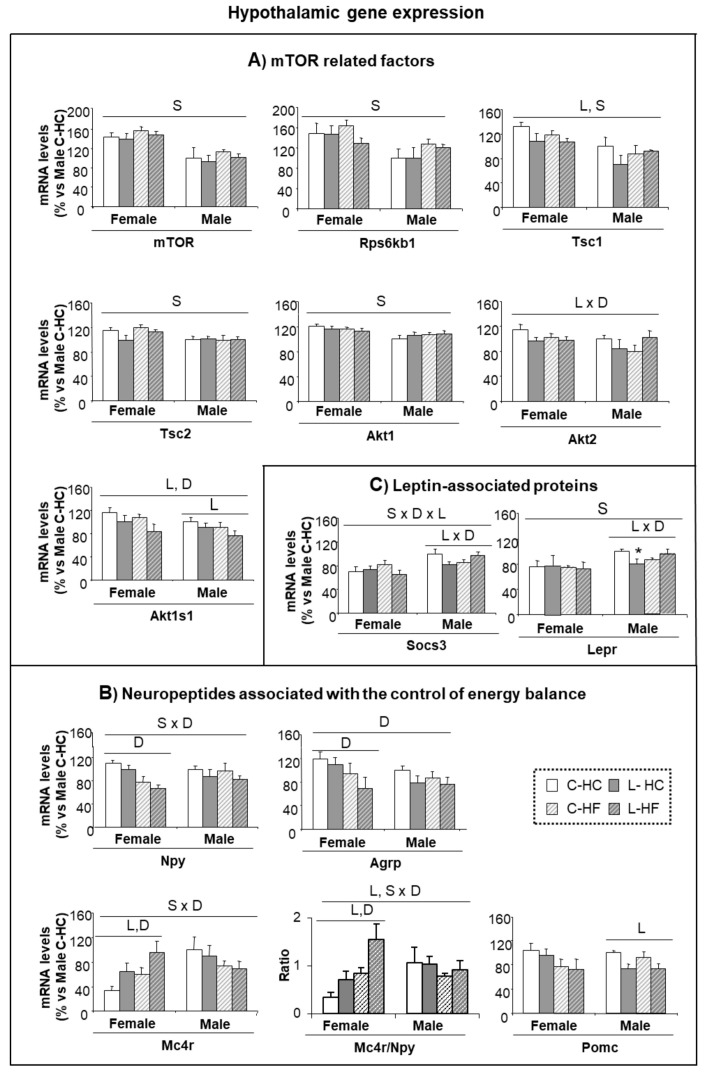
Hypothalamic gene expression in response to HF and HC diets. (**A**) Gene expression of mTOR related factors; (**B**) Expression of neuropeptides associated with the control of energy balance; and (**C**) Gene expression of leptin-associated proteins. Analyses were performed in C and Leu animals at the end of the HF/HC feeding (at nine months of age). mRNA levels have been analyzed by RT-PCR. All data represent mean ± SEM. The insert with the correspondence between the bar color and the experimental groups applies to (**A**,**B**,**C**) panels. Relative expression of control males (C-HC) has been set at 100% and used as a reference for the data of the rest of groups. Anova: S (sex), L (Leu), D (diet, HF vs. HC). * *p* < 0.05, student’s *t*-test.

The mTOR signaling pathway has been proposed as a central regulatory mechanism, particularly involved in glucose homeostasis and insulin sensitivity mediated by Leu, but also in protein and lipid metabolism [29]. Therefore, to check the impact of early Leu, an analysis of the gene expression of proteins and factors belonging to the mTOR pathway in the hypothalamus, at the end of the HF and HC diets, was performed (Figure 4A). In general, females presented higher levels of expression than males, and this was statistically significant for mTOR protein, its down-stream effector, the ribosomal kinase S6 kinase B1 (Rps6kb1), the regulatory proteins, tuberous sclerosis complex 1 (Tsc1) and 2 (Tsc2) and the RAC serine/threonine-protein kinase 1 (Akt1). In addition, early treatment with Leu induced a reduction in the expression of Tsc1 and in the proline-rich substrate of AKT1 (Akt1s1 also called PRAS40), the latter accentuated by HF-diet; a decreased expression of Akt2 was also found in L-animals under the HC-diet. No major differences were found in other proteins taking part in this signaling pathway (Figure S1A).

Relationships between the factors involved in the mTOR signaling pathway are complex and we did not determine the level of phosphorylation that could contribute to characterizing, with further detail, the adaptations in the mTOR pathway. However, it is remarkable that those factors affected in our experimental set up have also been associated with a model of activation of mTOR induced by insulin (AKT, TSC1 and 2 and PRAS40) and are relatively independent of the activation performed by amino acids [30]. The fact that females express higher levels than males, concomitant with a reduction in the expression of all these factors associated with early Leu supplementation is suggestive of altered insulin-dependent regulation of mTOR in the hypothalamus of L-females.

In order to assess whether early dysregulation found in food preferences affected neuropeptide regulation over the long-term, the expression of genes associated with the control of food intake was determined in the hypothalamus at the end of the HC/HF feeding. The expression of the orexigenic peptides—Npy and agouti related-peptide (Agrp)—showed the lowest levels in females under the HF diet, maybe as a reaction to counteract obesity progression (Figure 4B). Interestingly, expression of melanocortin 4 receptor (Mc4r) showed a sex-diet distinctive pattern; L-females under HC diet expressed more Mc4r than controls and this was accentuated under the HF diet, whereas males showed an opposed pattern.

Expression of the pro-opiomelanocortin (Pomc) anorexigenic peptide was lower in L-males in comparison with the controls and no differences were found within female groups (Figure 4B). In addition, the ratio of expression of Mc4r/Npy was similar in all male groups (close to 1) whereas in females the impacts of both early-Leu and the adult diet were underlined. Early-Leu supplementation was associated with a two-fold increase in the Mc4r/Npy ratio in females (2.0-fold and 1.8-fold under HF- and HC-diets, respectively), in comparison with the respective controls. Expression of the hypothalamic regulatory peptide, cocaine and amphetamine regulated transcript (Cartpt), signaling associated receptors, such as the growth hormone secretagogue receptor (Ghsr) and the insulin receptor (Insr), and functionally associated genes, like the fat mass obesity-associated protein (Fto) were neither different between genders, nor altered by treatments (Figure S1B). However, the influence of Leu supplementation was found, to some extent, on leptin signaling. Expression of the leptin receptor (Lepr) and of the suppressor of cytokine signaling 3 (Socs3) showed a similar pattern—they were lower in females than males and showed a decrease in L-males under HC-feeding, whereas a lack of response was observed in female groups (Figure 4C). 

**Figure 5:**
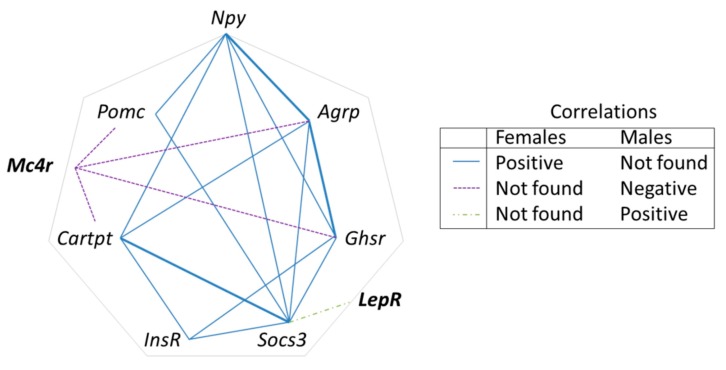
Pattern of correlations within hypothalamic neuropeptide expression in Leu-females in comparison with Leu-males. Linear relationships between the expressions of hypothalamic peptides were tested using Pearson’s correlation coefficients. Statistically significant correlations (*p* < 0.05) found are depicted. A continuous line between two peptides denotes the existence of a positive correlation in their expression in L-females, which was not present in L-males. A dotted line indicates that a negative correlation was found in L-males, which was not observed in L-females and a dashed-dotted line indicates that a positive correlation was found in L-males, which was not observed in L-females. Bold line shows the lack of response to Leu seen in females. The full set of data is shown in Table S2.

In connection with the different gender susceptibility to obesity found in L animals, a regression analysis revealed the existence of relationships within hypothalamic neuropeptide expressions in males, which were lost or altered in female-Leu animals. The whole set of correlations analysed is presented in Table S2. A scheme outlining the significant correlations and their relationships is shown in Figure 5. Mc4r expression in L-males was negatively correlated with Pomc, Cartpt, Agrp, and Ghsr, whereas such correlations were not found in L-females (Figure 5). The same pattern was observed concerning the expression of LepR and Socs3. On the contrary, a high number of positive correlations were seen in L-females, which were not observed in L-males (Figure 5), mostly concerning relationships between Npy and Socs3 with the rest of neuropeptides. Special relevance may exist for the correlations between Npy and Agrp, Agrp and Gshr and Socs3 and Cartpt, which were found in control animals, both male and females, and in L-females, but not in L-males. Therefore, these neuropeptides could be more directly involved in the protective effect against obesity seen in males.

Together, the data indicate that leucine supplementation in early life seems to disrupt—to some extent and particularly in females—the hypothalamic regulatory nexus driven by neuropeptide components, involved in the control of energy balance and fat stores. In contrast, the same nutritional input in male siblings reinforced the control of energy balance and over the long-term, protected, to some extent, animals, against the development of dietary obesity.

### 3.5. Selected Early Biomarkers at Weaning May Allow Identification of Susceptibility to Obesity in Adulthood

**Figure 6:**
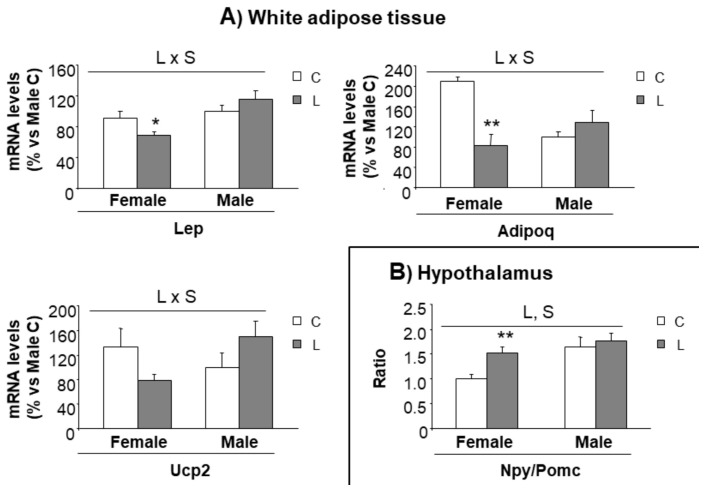
Expression of genes in the adipose tissue and hypothalamus at weaning. Expression of genes in (**A**) mesenteric adipose tissue and (**B**) hypothalamus at weaning in offspring from C and Leu-supplemented dams. mRNA have been analysed by RT-PCR. All data represent mean ± SEM. Relative expression of control males has been set at 100% and used to refer the data of the rest of groups. Anova: S (sex), L (Leu). * *p* < 0.05, ** *p* < 0.01, student’s *t*-test. Lep = leptin; Adipoq = Adiponectin; Ucp2 = uncoupling protein 2; Npy = Neuropeptide Y; Pomc = Pro-opiomelanocortin.

In order to track whether metabolic alterations found in adulthood could be anticipated at an earlier age, our next step was to further characterize gene expression in selected tissues (hypothalamus and mesenteric adipose tissue) of the animals at weaning, when offspring from control and Leu-dams still did not differentiate for body weight or body composition. Following the same pattern, the weight of mesenteric adipose tissue was not different between groups (Table S3). Interestingly, an altered pattern of gene expression could be found in adipose. Leptin (25%) and adiponectin (60%), key genes associated with energy metabolism, showed decreased expression in L-females (versus C-females), and the same trend was observed in Ucp2 expression (40%) (Figure 6A); profile in high contrast with the expression observed within males, which was not altered by early Leu treatment. This suggested that these genes could be earlier indicators of a gender-specific perinatal imprinting, associated with the susceptibility to develop obesity in adulthood.

**Figure 7:**
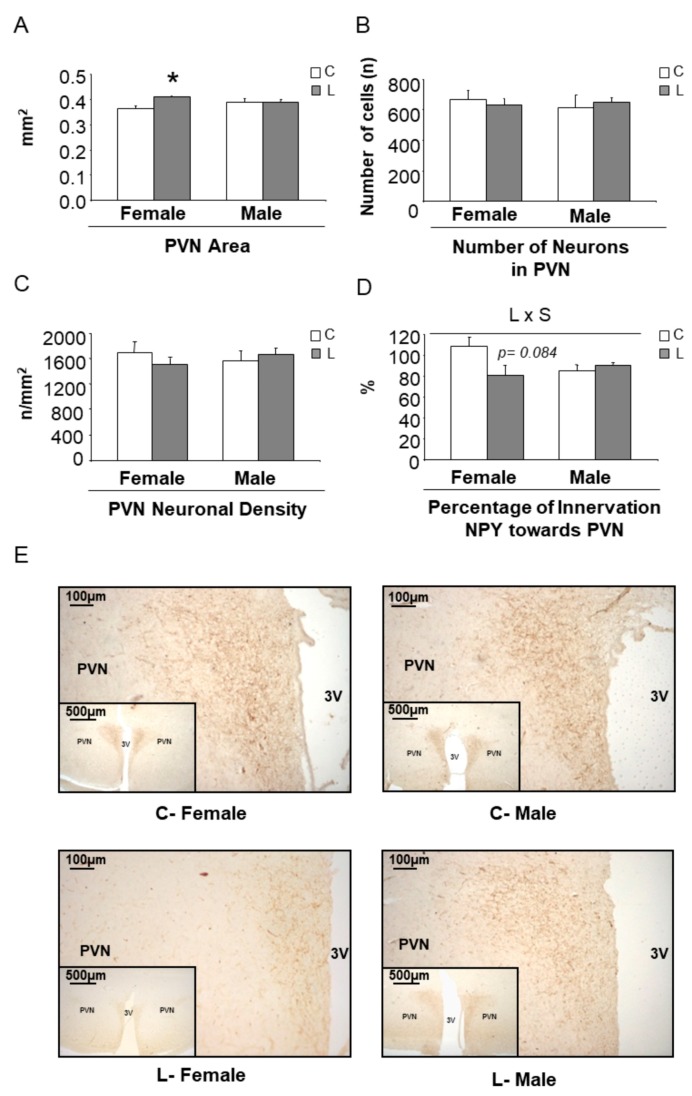
Morphometry of the paraventricular nucleus (PVN) and immunohistochemistry of NPY+ fibers at weaning. (**A**) PVN area, (**B**) number of neurons, (**C**) neuronal density, (**D**) innervation of neuropeptide Y (NPY+) fibers towards PVN and (**E**) representative brain sections immunostained for NPY in PVN. All data are from offspring (21 days of age) obtained from dams supplemented with Leu (L) during lactation and the corresponding sex-controls (**C**). All data represent mean ± SEM. * *p* < 0.05, student’s *t*-test.

Concerning the expression of hypothalamic factors associated with mTOR signaling (Figure S2A) and the expression of neuropeptides (Figure S2B), only minor effects were seen at this early stage. However, a higher Npy/Pomc ratio was found in L-females (versus C-females), whereas it was not altered in L-males (versus C-males) (Figure 6B), suggesting a tendency to higher orexigenic versus anorexigenic modulation, induced by maternal Leu-supplementation in female offspring. This fact, together with the disturbed expression of neuropeptides observed in adult life, directed our focus to NPY neuronal connections at weaning. The hypothalamic paraventricular nucleus (PVN) is the main site of accumulation of nerve terminals of NPY-containing neurons. A morphometric analysis of that region was performed and NPY positive (NPY+) axons were located by immunohistochemistry (Figure 7E). Data showed that female progeny from Leu-supplemented dams had a wider PVN area than their corresponding controls, whereas no differences were found within males (Figure 7A). The number of neurons in the PVN was similar between all experimental groups (Figure 7B) and the same was seen concerning PVN neuronal density (Figure 7C). However, a lower percentage of innervation of NPY+ fibers towards PVN was found in L-females, in comparison with controls, whereas no differences were seen in males, irrespective of maternal Leu supplementation (Figure 7D). These data show the impact of maternal Leu-supplementation in disturbing neuroanatomical development in offspring, which would be associated with inadequate NPY signaling in the central nervous system from early life, resulting in sustained disturbances in adulthood, in female animals.

## 4. Discussion

Obesity is growing at an alarming rate and has been described as one of the most serious public health challenges in Europe, particularly because of its increasing prevalence in children [1]. However, effective strategies to counteract and/or prevent obesity are still lacking. Only in a few particular cases do we know about the mechanism involved and have the specific tools to resolve the problem (i.e., in the case of leptin deficiency, which affects a small number of individuals, obesity can be treated with leptin injections [31]). Although, there are already some studies showing that the intake of specific nutrients at early life exerts important effects later in adulthood, as has been shown for leptin [32,33], a protein present in breast milk but not in infant formula [34] and for β-carotene [35]. Therefore, despite the identification of specific nutrients still being scarce, it is becoming clear that early nutrition constitutes a first line of intervention and that current evidence suggests dietary influence during pregnancy and lactation as main determinants of obesity in offspring in both animal models and humans [32,36,37,38,39,40,41,42]. Here, we show that in female rats, maternal leucine supplementation during lactation predisposes offspring, when adults, to higher body fat accumulation, whereas it protects male offspring.

Our approach started from the observation that breast milk contains soluble, free amino acids that are lost in commercial milks for infants [43,44] and the consideration that this could have a noticeable role in short term modulation of nutritional cues in the gastrointestinal tracts of neonates. In this context, we made an intervention in the amount of Leu, a single and essential component in the maternal diet, taking advantage of the fact that Leu is an indispensable amino acid and therefore, its levels are exclusively dependent on dietary sources. Leu was selected because of its high abundance in body proteins and fluids, including milk, where it has been involved in the growth-promoting effects of milk [45]. Recently, the current trend towards obesity found in non-breast fed infants has been associated with the highest protein levels in commercial milk formulas, which are elaborated with cow proteins, richer in Leu than those of humans. In that situation, Leu exacerbation of mTOR signaling could be the linking mechanism between early feeding and adult obesity [46]. However, the daily amount of Leu required to support normal body functions and its role in body weight control is not clear at all. As an example, the setting up of Leu recommendations in growing infants is based on the Leu-protein content in breast milk, but does not take into account the significant amount of free Leu found in breast milk [47], so may be underestimating potential needs. Furthermore, some authors highlight that requirements for Leu in adults should be increased [48] and that higher daily intakes could contribute to a leaner phenotype, particularly in weight loss diets [11,49,50,51,52]. Moreover, there is controversy on the beneficial role of Leu in body fat accretion and glucose/insulin homeostasis. There is evidence supporting the improvement of these metabolic signatures [15,19,53,54,55,56], at least in certain conditions, but also a lack of effects has been found, which appears mainly associated with low doses, short treatment times or the fact that treatment was performed in non-obese animals [17,57,58]. 

Recently, a collection of papers showing fat mobilization and weight loss in animals after short-term deprivation of Leu introduced a novel perspective in this area [19,20,21,23]. In the previous characterization of maternal metabolism, we found that dietary Leu supplementation during lactation was associated with a relative improvement in body composition [24]. A basic question was whether this beneficial influence could be transferred towards progeny. Our data were consistent with normal growth rates and development of progeny, although this went together with a lower food intake in females that was not observed in males. However, when a food preference test was performed and animals were able to select between a fat-rich (olive oil emulsion) or sucrose-rich drink, perturbations in underlying mechanisms of food intake and selection became apparent. Particularly, a decrease in sucrose preference was found and preference for fat remained constant, a trait that could contribute to a predisposition towards a higher calorie intake during hyperlipidic diets. Then, the challenge with a HF diet allowed us to provide evidence for the presence of disturbances in the regulatory axis, controlling food intake, body weight and neuroendocrine signaling in hypothalamus. The high-fat diet, in L-females, initially caused a higher food intake, associated with a higher degree of obesity, which was already apparent after 15 days of the diet, in comparison with a more delayed and smoother response in the respective C-females. Interestingly, L-males did not show the same pattern and in fact, they appeared to be somehow protected against body fat accretion, either under a high-fat or a high-sucrose diet (around 15% less body fat). These results may contribute to unravelling the controversial role of Leu supplementation, which in addition to having opposite effects depending on sex, timing of administration and impact at long-term appear to also be key factors that should be taken into account.

Finally, in our model based on increased maternal consumption of Leu during lactation and its withdrawal at weaning, a sex-specific effect was found in the offsprings’ insulin homeostasis. Gender-dependent differences were found for insulin levels (higher in males than in females), already present in C-animals, which was in accordance with other previous publications. Moreover, maternal consumption of Leu during lactation differentially affected the response to an OGTT within each sex [42,59,60]. Specifically, control of glycaemia after the OGTT was associated with higher insulin release in Leu-females in comparison with C-females; whereas in males, the opposing pattern was observed. Consequently, early Leu supplementation was associated with improved insulin sensitivity in males in contrast with the adverse impact on females. Recently, coordinated and parallel pathways regulating mTOR by insulin and amino acids (mainly Leu) have been described. Activation of the mTOR pathway through the complex mTOR-Raptor modulates the translation of specific mRNA, mediated by the ribosomal kinase S6K1 and the binding protein to the initiation factor of protein synthesis 4E-BP1 [30]; mTOR and S6K1 are two factors that showed a higher expression in females than in males in our study. In addition, perinatal treatment with Leu modified the expression of *Akt* and of the target proteins of its phosphorylation, *Pras40* and *Tsc*, which could be associated with a diminution of this signaling pathway by Leu in general and may be more evident during high-fat feeding. The mTOR pathway is at metabolic confluence with the nutrient-hormonal signaling network and integrates with the energy status of cells, therefore, its dysregulation has been implicated in the development of obesity [61]. Therefore, perturbations found in the expression of relevant genes in this regulatory pathway could be at the basis of sex-specific different susceptibilities to obesity associated with Leu.

In close connection with this, hypothalamic anatomical development and the associated neuropeptide expression network, which drive the regulation of energy balance, were also disturbed, as described in other animal models of nutritional manipulation during the perinatal period [59,62]. The relevance of NPY and its role, in combination with other regulatory peptides (POMC, AgRP, MC4-R and SOCS3), was clearly seen in adulthood, when obesity was settled in L-females. Interestingly, susceptibility to obesity could also be anticipated at weaning from hypothalamic and adipose tissue metabolic signatures. The identification of early biomarkers of adult health outcomes is a remarkable interest of obesity research. Such indicators may provide a useful tool to assess susceptibility to obesity in advance and therefore, contribute to delaying its onset and/or counteracting its development [32,63,64]. 

## 5. Conclusions

This study demonstrates that maternal Leu supplementation during lactation contributes to inducing a developmental program which predisposes female offspring to higher susceptibility to adult dietary obesity, while protecting male sibling offspring. The molecular basis of the disturbance is likely to be linked to neuropeptide expression, with a relevant role for *Npy*, and mTOR factors associated with insulin signaling. Early warning signatures of adverse nutritional imprinting could be anticipated from leptin and adiponectin expression in adipose tissue, long before the onset of obesity. 

## Supplementary Materials

The following are available online at http://www.mdpi.com/2072-6643/10/1/76/s1, Figure S1: Hypothalamic gene expression at adulthood, Figure S2: Expression of genes in hypothalamus at weaning. Table S1: Nucleotide sequences of primers used for PCR amplification; Table S2: Correlations within hypothalamic neuropeptide expression in progeny of C and Leu-supplemented dams. Table S3: Weight of mesenteric white adipose tissue (mWAT) at weaning.

## References

[1] Branca F., Nikogosian H., Lobstein T. (2007). The Challenge of Obesity in the WHO European Region and the Strategies for Response.

[2] Macotela Y., Emanuelli B., Bång A.M., Espinoza D.O., Boucher J., Beebe K., Gall W., Kahn C.R. (2011). Dietary leucine—An environmental modifier of insulin resistance acting on multiple levels of metabolism. PLoS ONE.

[3] Cottrell E.C., Ozanne S.E. (2008). Early life programming of obesity and metabolic disease. Physiol. Behav..

[4] Bouret S.G. (2009). Early life origins of obesity: Role of hypothalamic programming. J. Pediatr. Gastroenterol. Nutr..

[5] Carlson S.E. (2009). Early determinants of development: A lipid perspective. Am. J. Clin. Nutr..

[6] Pico C., Palou A. (2013). Perinatal programming of obesity: An introduction to the topic. Front. Physiol..

[7] Caspó J., Salamon S. (2009). Composition of the mother’s milk i. Protein contents, amino acid composition, biological value. A review. Acta Univ. Sapientiae Aliment..

[8] Chavalittamrong B., Suanpan S., Boonvisut S., Chatranon W., Gershoff S.N. (1981). Protein and amino acids of breast milk from thai mothers. Am. J. Clin. Nutr..

[9] Pedroso J.A., Zampieri T.T., Donato J. (2015). Reviewing the effects of l-leucine supplementation in the regulation of food intake, energy balance, and glucose homeostasis. Nutrients.

[10] Layman D.K. (2003). The role of leucine in weight loss diets and glucose homeostasis. J. Nutr..

[11] Shah S.H., Crosslin D.R., Haynes C.S., Nelson S., Turer C.B., Stevens R.D., Muehlbauer M.J., Wenner B.R., Bain J.R., Laferrère B. (2012). Branched-chain amino acid levels are associated with improvement in insulin resistance with weight loss. Diabetologia.

[12] Krebs M., Krssak M., Bernroider E., Anderwald C., Brehm A., Meyerspeer M., Nowotny P., Roth E., Waldhäusl W., Roden M. (2002). Mechanism of amino acid-induced skeletal muscle insulin resistance in humans. Diabetes.

[13] Rachdi L., Aïello V., Duvillié B., Scharfmann R. (2012). L-leucine alters pancreatic β-cell differentiation and function via the mtor signaling pathway. Diabetes.

[14] Li F., Yin Y., Tan B., Kong X., Wu G. (2011). Leucine nutrition in animals and humans: Mtor signaling and beyond. Amino Acids.

[15] Zhang Y., Guo K., LeBlanc R.E., Loh D., Schwartz G.J., Yu Y.H. (2007). Increasing dietary leucine intake reduces diet-induced obesity and improves glucose and cholesterol metabolism in mice via multimechanisms. Diabetes.

[16] Yao K., Duan Y., Li F., Tan B., Hou Y., Wu G., Yin Y. (2016). Leucine in obesity: Therapeutic prospects. Trends Pharmacol. Sci..

[17] Nairizi A., She P., Vary T.C., Lynch C.J. (2009). Leucine supplementation of drinking water does not alter susceptibility to diet-induced obesity in mice. J. Nutr..

[18] Lynch C.J., Hutson S.M., Patson B.J., Vaval A., Vary T.C. (2002). Tissue-specific effects of chronic dietary leucine and norleucine supplementation on protein synthesis in rats. Am. J. Physiol. Endocrinol. Metab..

[19] Cheng Y., Meng Q., Wang C., Li H., Huang Z., Chen S., Xiao F., Guo F. (2010). Leucine deprivation decreases fat mass by stimulation of lipolysis in white adipose tissue and upregulation of uncoupling protein 1 (ucp1) in brown adipose tissue. Diabetes.

[20] Cheng Y., Zhang Q., Meng Q., Xia T., Huang Z., Wang C., Liu B., Chen S., Xiao F., Du Y. (2011). Leucine deprivation stimulates fat loss via increasing crh expression in the hypothalamus and activating the sympathetic nervous system. Mol. Endocrinol..

[21] Xiao F., Huang Z., Li H., Yu J., Wang C., Chen S., Meng Q., Cheng Y., Gao X., Li J. (2011). Leucine deprivation increases hepatic insulin sensitivity via gcn2/mtor/s6k1 and ampk pathways. Diabetes.

[22] Xia T., Cheng Y., Zhang Q., Xiao F., Liu B., Chen S., Guo F. (2012). S6k1 in the central nervous system regulates energy expenditure via mc4r/crh pathways in response to deprivation of an essential amino acid. Diabetes.

[23] Zhang Q., Liu B., Cheng Y., Meng Q., Xia T., Jiang L., Chen S., Liu Y., Guo F. (2014). Leptin signaling is required for leucine deprivation-enhanced energy expenditure. J. Biol. Chem..

[24] López N., Sánchez J., Picó C., Palou A., Serra F. (2010). Dietary l-leucine supplementation of lactating rats results in a tendency to increase lean/fat ratio associated to lower orexigenic neuropeptide expression in hypothalamus. Peptides.

[25] Sánchez J., Priego T., Palou M., Tobaruela A., Palou A., Picó C. (2008). Oral supplementation with physiological doses of leptin during lactation in rats improves insulin sensitivity and affects food preferences later in life. Endocrinology.

[26] Hsu S.M., Raine L., Fanger H. (1981). Use of avidin-biotin-peroxidase complex (abc) in immunoperoxidase techniques: A comparison between abc and unlabeled antibody (pap) procedures. J. Histochem. Cytochem..

[27] Sclafani A. (2002). Flavor preferences conditioned by sucrose depend upon training and testing methods: Two-bottle tests revisited. Physiol. Behav..

[28] Takeda M., Imaizumi M., Fushiki T. (2000). Preference for vegetable oils in the two-bottle choice test in mice. Life Sci..

[29] Lamming D.W., Sabatini D.M. (2013). A central role for mtor in lipid homeostasis. Cell Metab..

[30] Dennis M.D., Baum J.I., Kimball S.R., Jefferson L.S. (2011). Mechanisms involved in the coordinate regulation of mtorc1 by insulin and amino acids. J. Biol. Chem..

[31] Frank S., Heni M., Moss A., von Schnurbein J., Fritsche A., Häring H.U., Farooqi S., Preissl H., Wabitsch M. (2011). Leptin therapy in a congenital leptin-deficient patient leads to acute and long-term changes in homeostatic, reward, and food-related brain areas. J. Clin. Endocrinol. Metab..

[32] Szostaczuk N., Priego T., Palou M., Palou A., Picó C. (2017). Oral leptin supplementation throughout lactation in rats prevents later metabolic alterations caused by gestational calorie restriction. Int. J. Obes..

[33] Konieczna J., García A.P., Sánchez J., Palou M., Palou A., Picó C. (2013). Oral leptin treatment in suckling rats ameliorates detrimental effects in hypothalamic structure and function caused by maternal caloric restriction during gestation. PLoS ONE.

[34] Resto M., O’Connor D., Leef K., Funanage V., Spear M., Locke R. (2001). Leptin levels in preterm human breast milk and infant formula. Pediatrics.

[35] Mušinović H., Bonet M.L., Granados N., Amengual J., von Lintig J., Ribot J., Palou A. (2014). B-carotene during the suckling period is absorbed intact and induces retinoic acid dependent responses similar to preformed vitamin a in intestine and liver, but not adipose tissue of young rats. Mol. Nutr. Food Res..

[36] Picó C., Palou M., Priego T., Sánchez J., Palou A. (2012). Metabolic programming of obesity by energy restriction during the perinatal period: Different outcomes depending on gender and period, type and severity of restriction. Front. Physiol..

[37] Heerwagen M.J., Miller M.R., Barbour L.A., Friedman J.E. (2010). Maternal obesity and fetal metabolic programming: A fertile epigenetic soil. Am. J. Physiol. Regul. Integr. Comp. Physiol..

[38] Waterland R.A., Jirtle R.L. (2004). Early nutrition, epigenetic changes at transposons and imprinted genes, and enhanced susceptibility to adult chronic diseases. Nutrition.

[39] Waterland R.A., Travisano M., Tahiliani K.G., Rached M.T., Mirza S. (2008). Methyl donor supplementation prevents transgenerational amplification of obesity. Int. J. Obes..

[40] Palou M., Priego T., Romero M., Szostaczuk N., Konieczna J., Cabrer C., Remesar X., Palou A., Pico C. (2015). Moderate calorie restriction during gestation programs offspring for lower bat thermogenic capacity driven by thyroid and sympathetic signaling. Int. J. Obes..

[41] Castro H., Pomar C.A., Palou A., Picó C., Sánchez J. (2017). Offspring predisposition to obesity due to maternal-diet-induced obesity in rats is preventable by dietary normalization before mating. Mol. Nutr. Food Res..

[42] Pomar C.A., van Nes R., Sánchez J., Picó C., Keijer J., Palou A. (2017). Maternal consumption of a cafeteria diet during lactation in rats leads the offspring to a thin-outside-fat-inside phenotype. Int. J. Obes..

[43] Chuang C.K., Lin S.P., Lee H.C., Wang T.J., Shih Y.S., Huang F.Y., Yeung C.Y. (2005). Free amino acids in full-term and pre-term human milk and infant formula. J. Pediatr. Gastroenterol. Nutr..

[44] Agostoni C., Carratù B., Boniglia C., Riva E., Sanzini E. (2000). Free amino acid content in standard infant formulas: Comparison with human milk. J. Am. Coll. Nutr..

[45] Millward D.J. (2012). Knowledge gained from studies of leucine consumption in animals and humans. J. Nutr..

[46] Melnik B.C. (2012). Excessive leucine-mtorc1-signalling of cow milk-based infant formula: The missing link to understand early childhood obesity. J. Obes..

[47] World Health Organization (2007). Protein and Amino Acid Requirements in Human Nutrition.

[48] Elango R., Ball R.O., Pencharz P.B. (2008). Individual amino acid requirements in humans: An update. Curr. Opin. Clin. Nutr. Metab. Care.

[49] Layman D.K. (2004). Protein quantity and quality at levels above the rda improves adult weight loss. J. Am. Coll. Nutr..

[50] Layman D.K., Evans E.M., Erickson D., Seyler J., Weber J., Bagshaw D., Griel A., Psota T., Kris-Etherton P. (2009). A moderate-protein diet produces sustained weight loss and long-term changes in body composition and blood lipids in obese adults. J. Nutr..

[51] Devkota S., Layman D.K. (2010). Protein metabolic roles in treatment of obesity. Curr. Opin. Clin. Nutr. Metab. Care.

[52] Mojtahedi M.C., Thorpe M.P., Karampinos D.C., Johnson C.L., Layman D.K., Georgiadis J.G., Evans E.M. (2011). The effects of a higher protein intake during energy restriction on changes in body composition and physical function in older women. J. Gerontol. A Biol. Sci. Med. Sci..

[53] Binder E., Bermúdez-Silva F.J., André C., Elie M., Romero-Zerbo S.Y., Leste-Lasserre T., Belluomo L., Duchampt A., Clark S., Aubert A. (2013). Leucine supplementation protects from insulin resistance by regulating adiposity levels. PLoS ONE.

[54] Li H., Xu M., Lee J., He C., Xie Z. (2012). Leucine supplementation increases sirt1 expression and prevents mitochondrial dysfunction and metabolic disorders in high-fat diet-induced obese mice. Am. J. Physiol. Endocrinol. Metab..

[55] Eller L.K., Saha D.C., Shearer J., Reimer R.A. (2013). Dietary leucine improves whole-body insulin sensitivity independent of body fat in diet-induced obese sprague-dawley rats. J. Nutr. Biochem..

[56] Chen H., Simar D., Ting J.H., Erkelens J.R., Morris M.J. (2012). Leucine improves glucose and lipid status in offspring from obese dams, dependent on diet type, but not caloric intake. J. Neuroendocrinol..

[57] Noatsch A., Petzke K.J., Millrose M.K., Klaus S. (2011). Body weight and energy homeostasis was not affected in c57bl/6 mice fed high whey protein or leucine-supplemented low-fat diets. Eur. J. Nutr..

[58] Freudenberg A., Petzke K.J., Klaus S. (2012). Comparison of high-protein diets and leucine supplementation in the prevention of metabolic syndrome and related disorders in mice. J. Nutr. Biochem..

[59] Palou M., Torrens J.M., Priego T., Sánchez J., Palou A., Picó C. (2011). Moderate caloric restriction in lactating rats programs their offspring for a better response to HF diet feeding in a sex-dependent manner. J. Nutr. Biochem..

[60] Palou M., Konieczna J., Torrens J.M., Sánchez J., Priego T., Fernandes M.L., Palou A., Picó C. (2012). Impaired insulin and leptin sensitivity in the offspring of moderate caloric-restricted dams during gestation is early programmed. J. Nutr. Biochem..

[61] Catania C., Binder E., Cota D. (2011). Mtorc1 signaling in energy balance and metabolic disease. Int. J. Obes..

[62] Palou M., Priego T., Sánchez J., Palou A., Picó C. (2010). Sexual dimorphism in the lasting effects of moderate caloric restriction during gestation on energy homeostasis in rats is related with fetal programming of insulin and leptin resistance. Nutr. Metab..

[63] Konieczna J., Sánchez J., van Schothorst E.M., Torrens J.M., Bunschoten A., Palou M., Picó C., Keijer J., Palou A. (2014). Identification of early transcriptome-based biomarkers related to lipid metabolism in peripheral blood mononuclear cells of rats nutritionally programmed for improved metabolic health. Genes Nutr..

[64] Torrens J.M., Konieczna J., Palou M., Sánchez J., Picó C., Palou A. (2014). Early biomarkers identified in a rat model of a healthier phenotype based on early postnatal dietary intervention may predict the response to an obesogenic environment in adulthood. J. Nutr. Biochem..

