# Supplementary material for: Gender-Associated Impact of Early Leucine Supplementation on Adult Predisposition to Obesity in Rats
Source: Nutrients. 2018 Jan 12;10(1):76. doi: 10.3390/nu10010076 (PMC5793304; doi:10.3390/nu10010076)
Supplement: Supplementary file 1 [file nutrients-10-00076-s001.pdf]

Table S1 Nucleotide sequences of primers used for PCR amplification.

Gene	Forward Primer (5' to 3')	Reverse Primer (5' to 3')	Amplicon size (bp)
18S	CGCGGTTCTATTTTGTTGGT	AGTCGGCATCGTTTATGGTC	219
Eif4ebp1	CAAAACACCCCCAAAGGAC	CACCTGCCCGCTTATCTTC	122
Adipoq	GCTCAGGATGCTACTGTTG	TCTCACCCTTAGGACCAAG	247
Agrp	AGAGTTCTCAGGTCTAAGTCT	CTTGAAGAAGCGGCAGTAGCACGT	210
Akt1	ACTCATTCCAGACCCACGAC	AGCCCGAAGTCCGTTATCTT	242
Akt2	ATGATGGAGGTAGCGGTCAG	TGTAGGACTCGGCTCTCTGTG	212
Cartpt	AGAAGAAGTACGGCCAAGTCC	CACACAGCTTCCCGATCC	84
Fto	CAGCAGTGGCAGCTGAAATA	CAGCAGTGGCAGCTGAAATA	180
Ghsr	TCAGCCAGTACTGCAACCTG	GGAGAGATGGGATGTGCTGT	222
Insr	CTCCTGGGATTCATGCTGTT	GTCCGGCGTTCATCAGAG	242
Lep	TTCACACACGCAGTCGGTAT	AGGTCTCGCAGGTTCTCCAG	186
Mc4r	TATGGTACTGGAGCGCGTAA	TCAGACGGAGGATGCTATGA	370
mTOR	CCCAGCCCTACAAAGTCCA	ACCTCACCGCCACAGAAAG	292
Npy	TGGACTGACCCTCGCTCTAT	GTGTCTCAGGGCTGGATCTC	188
Lepr	TGAAACATTTGAGCATCTTT	CGATGCACTGGCTGACAGAA	368
Pik3c3	TGGTTGAGTTTCGCTGTGTC	CTTGTGGTGTTTGCTCTCCA	164
Pomc	CCTGTGAAGGTGTACCCCAATGTC	CACGTTCTTGATGATGGCGTTC	266
Pras40	CAAGGAGAAGAGGACAGAAGC	GAAAGTCGCTGGTATTGAGC	170
Rptor	CCTACGGTGAATGGAGAGGT	ATCTGGGCAAGTGGATGGT	221
Rheb	GTTATCCACGGCAAGCTGTT	GCTCCGTCAATCTTTTCTGC	227
Rragb	ATTCCCCAGAAGCCAAAA	CAGGTTCATTTCCAGTTGCT	227
S6k1	GACATGGCAGGAGTGTTTGA	TTTCCATAGCCCCCTTTACC	245
Socs3	TTCGGGACTAGGTAGGAAGGA	AGGGCCCCAGTCTGAGTATT	123
Tsc1	TTATCCATCCTCTCGCTGCT	AGGTGCTGCTTCCCTGACT	194
Tsc2	ATGGATGTTGGCTTGTCCTC	CAGGCAGTTGTAGCAGACCA	192
Ucp2	GGTCGGAGATACCAGAGCAC	ATGAGGTTGGCTTTCAGGAG	174
β-actin	TACAGCTTCACCACCACAGC	TCTCCAGGGAGGAAGAGGAT	120

Abbreviations: 18s, Ribosomic RNA 18s; Eif4ebp1, Eukaryotic Translation Initiation Factor 4E Binding Protein 1; Adipoq, Adiponectin; Agrp, Agouti related peptide; Akt1, V-akt murine thymoma viral oncogene homolog 1; Akt2, V-akt murine thymoma viral oncogene homolog 2; Cartpt, CART Prepropeptide; Fto, Fat mass and obesity-associated protein; Ghsr, Ghrelin receptor; Insr, Insulin receptor; Lep, Leptin; Mcr4, Melanocortin 4 receptor; mTOR, Mechanistic Target of Rapamycin Kinase; Npy, Neuropeptide Y; Lepr, Leptin receptor; Pik3c3, Phosphatidylinositol-4,5-Bisphosphate 3-Kinase Catalytic Subunit Alpha; Pomc, Pro-opiomelanocortin; Pras40, Proline-rich AKT1 substrate of 40 kDa; Rptor, Regulatory associated protein of mTOR complex; Rheb, Ras homolog enriched in brain; Rragb, Ras-related GTP binding B; S6k1, Ribosomal protein S6 kinase; Socs3, Suppressor of cytokine signaling 3; Tsc1, Tuberous sclerosis 1; Tsc2, Tuberous sclerosis 2; Ucp2, uncoupling protein 2; β-actin, Beta-actin.

Table S2. Correlations within hypothalamic neuropeptide expression in progeny of C and Leu-supplemented dams. The comparison was done by the Pearson Chi-square test. Statistical significance (2-tailed) $p < 0.05$ (*), $p < 0.01$ (**).

		Female		Male	
		Control	Leucine	Control	Leucine
Npy	*Socs3*	-0.208	0.668*	0.087	-0.375
	Agrp	0.847**	0.747**	0.613*	0.077
	Mcr4	-0.196	-0.363	-0.009	0.383
	Insr	0.655*	0.551	0.235	0.181
	Pomc	0.676*	0.769**	0.537	0.088
	Ghsr	0.767**	0.741**	0.451	-0.509
	Lepr	0.190	0.083	0.234	0.328
	Cartpt	-0.276	0.724**	0.449	-0.287
Socs3	*Agrp*	0.169	0.687*	0.448	0.198
	Mcr4	-0.044	0.132	0.318	-0.341
	Insr	-0.317	0.606*	0.117	0.085
	Pomc	0.188	0.647*	0.410	0.254
	Ghsr	0.178	0.776**	0.508	0.453
	Lepr	0.327	0.230	0.422	0.609*
	Cartpt	0.865**	0.830**	0.700*	0.531
Agrp	*Mcr4*	-0.030	0.099	-0.079	-0.723**
	Insr	0.647*	0.391	0.225	0.468
	Pomc	0.820**	0.835**	0.810**	0.912**
	Ghsr	0.895**	0.749**	0.729**	0.582
	Lepr	0.443	0.170	0.304	0.242
	Cartpt	-0.007	0.746**	0.736**	0.542
Mcr4	*Insr*	0.209	0.155	0.551	-0.145
	Pomc	-0.260	-0.008	0.091	-0.715**
	Ghsr	-0.032	0.180	-0.004	-0.911**

	Lepr	-0.111	-0.129	0.452	-0.372
	Cartpt	-0.288	0.161	0.148	-0.637*
Insr	*Pomc*	0.685*	0.483	0.397	0.465
	Ghsr	0.777**	0.639*	0.322	0.174
	Lepr	0.215	0.244	0.020	0.336
	Cartpt	-0.391	0.675*	0.258	0.256
Pomc	*Ghsr*	0.864**	0.780**	0.858**	0.732*
	Lepr	0.599*	0.031	0.059	0.300
	Cartpt	0.159	0.714**	0.744**	0.700**
Ghsr	*Lepr*	0.404	0.297	0.019	0.494
	Cartpt	0.077	0.905**	0.698*	0.741**
Lepr	*Cartpt*	0.245	0.210	0.455	0.513

Table S3. Weight of mesenteric white adipose tissue (mWAT) at weaning. All data represent mean ± SEM. Statistical differences between control and leucine animals were assessed by student's *t*-test (* $p< 0.05$).

	**Female**		**Male**	
	**Control**	**Leucine**	**Control**	**Leucine**
**mWAT *(g)***	0.209 ± 0.017	0.205 ± 0.013	0.211 ± 0.022	0.158 ± 0.014

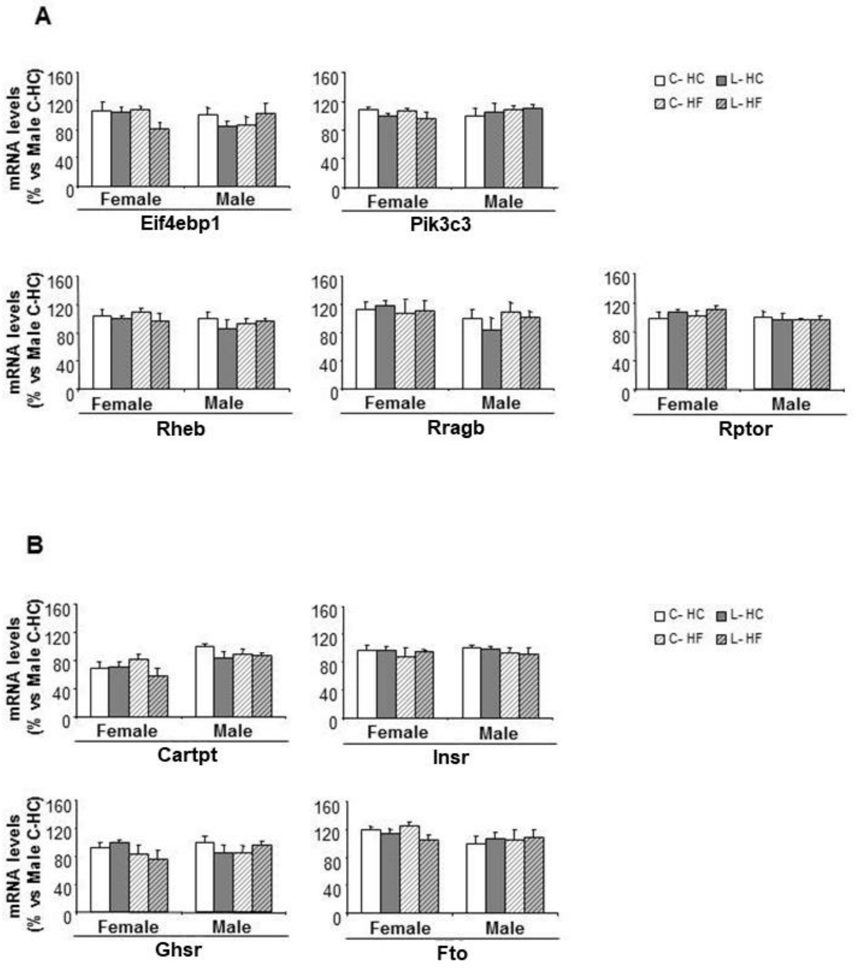

Figure S1. Hypothalamic gene expression (A) of mTOR related factors and (B) of genes associated with metabolism and energy balance, determined in C and Leu animals at the end of the HF/HC feeding (at 9 months of age). mRNA levels have been analysed by RT-PCR. All data represent mean ± SEM. Relative expression of control males (C-HC) has been set at 100% and used as a reference for the data of the rest of groups.

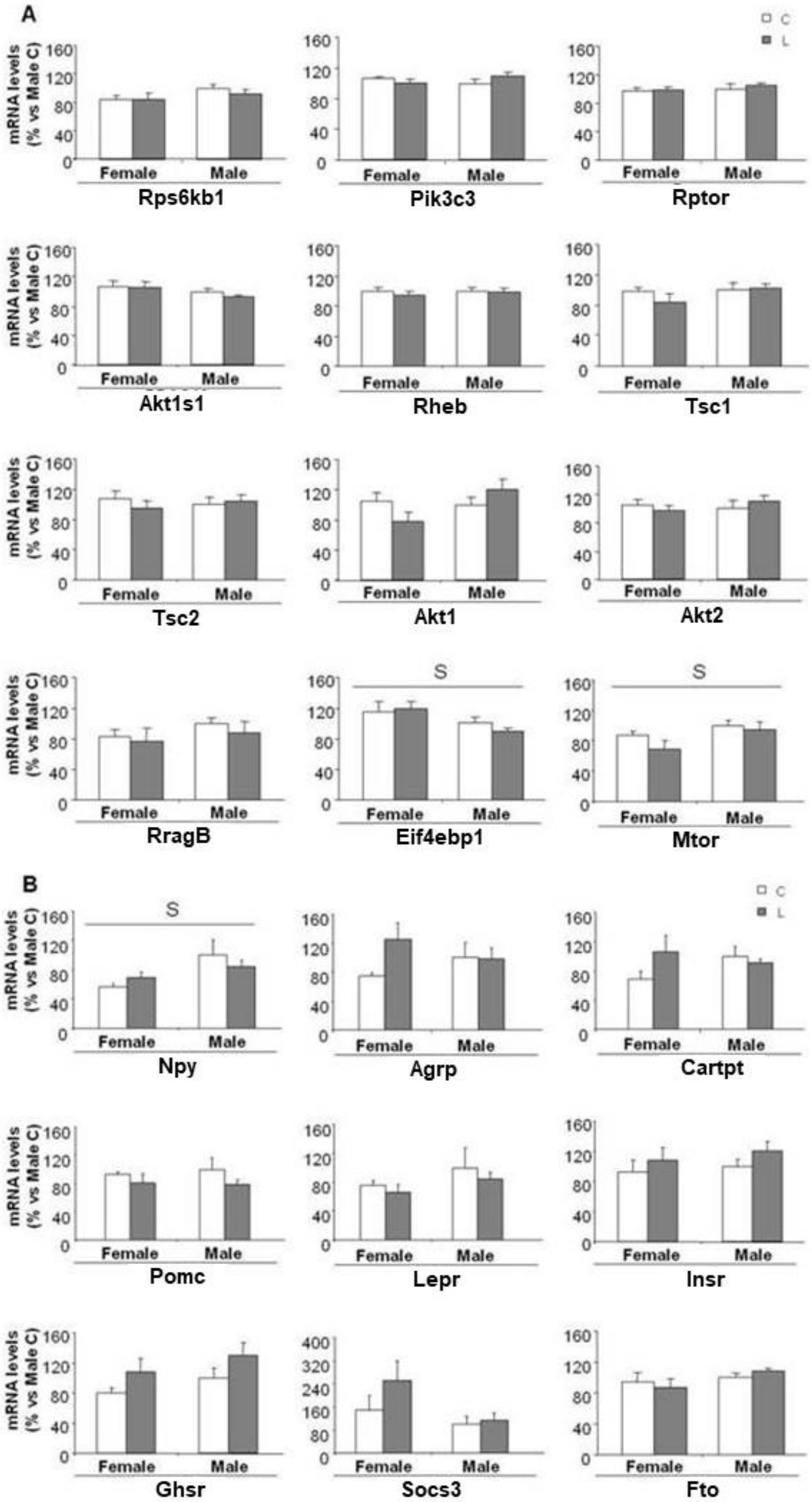

Figure S2. Expression of genes in hypothalamus at weaning (A) of mTOR pathway and (B) neuropeptides and other energy balance related proteins, at weaning in offspring from C and Leu-supplemented dams. mRNA have been analysed by RT-PCR. All data represent mean ± SEM. Relative expression of control males has been set at 100% and used as a reference for the data of the rest of groups. Anova: S (sex).
